# Prognostic Value of Microvessel Density in Tumor and Peritumoral Area as Evaluated by CD31 Protein Expression and Argyrophilic Nucleolar Organizer Region Count in Endothelial Cells in Uterine Leiomyosarcoma

**DOI:** 10.1155/2012/594512

**Published:** 2012-07-15

**Authors:** Ashot Avdalyan, Igor Bobrov, Vladimir Klimachev, Alexander Lazarev

**Affiliations:** ^1^Department of Pathology, Altai Branch of Blokhin Russian Cancer Research Center, Barnaul 656049, Russia; ^2^Department of Pathology, Altai State Medical University, Barnaul 656049, Russia

## Abstract

The objective of this study was to investigate the prognostic value of microvessel density (MVD) in uterine leiomyosarcoma (LMS) and peritumoral area (PA) as evaluated by CD31 expression and argyrophilic nucleolar organizer region (AgNOR) count in endothelial cells. Tissue specimens from 66 patients with uterine LMS were examined. There were no significant differences in the mean MVD between tumor itself and the PA (*P* = 0.9); moreover, the MVD in the PA often exceeded that in the tumor. No correlation or significant differences were also found in the MVD between different grades of malignancy of LMS (*r* = 0.1; *P* = 0.07). The number of AgNORs in tumor endothelial cells was significantly higher in tumor vessels than in the peritumoral area (*P* < 0.005) and increased with the tumor grade. Analysis of the prognostic value of MVD in uterine LMS and PA showed that the density of tumor vessels was not an independent criterion, while the MVD in the PA affected 10-year survival to a significantly greater extent (*χ* ^2^ = 27.5; *P* = 0.0003). The number of AgNORs also had an important effect on survival of LMS patients: when the threshold of 11.6 granules was exceeded, prognosis was significantly more unfavorable than that prior to exceeding the threshold.

## 1. Introduction

Uterine leiomyosarcoma (LMS) is a rare, highly malignant tumor of female reproductive system; according to different studies, its 5-year survival rate varies from 29% to 67.4% [1]. There are many data on the prognostic significance of several clinical and pathological parameters in tumors of the same location: stage, grade of malignancy, and some molecular biological criteria [2–5]. At the same time, the role of angiogenesis and the degree of maturity of microvessels in the prognosis of uterine leiomyosarcoma are of great interest; however, the literature data on this question are few and scattered [6].

A peritumoral area (PA) of any malignant tumor has a special biological role. It is this area that we keep in mind when speaking about “a tumor field area,” because in this area the tumor starts to grow and spread. Changes in tissues not involved in malignant growth are of interest for detecting background processes contributing to tumor growth. The peritumoral area can be characterized by a number of quantitative measures considering morphological characteristics of histohematic barrier, patterns of proliferation of cell populations, and their relationships with each other, including elements of microvasculature [7].

Angiogenesis is essential for the growth and metastatic dissemination of tumors of various locations. At the same time, it is well known that the antiangiogenic therapy (including targeted therapy, e.g., Avastin) can be used to inhibit tumor neoangiogenesis [8–11]. However, the effect of peritumoral vessel growth on the tumor outcome has been poorly studied [12–14]. There is evidence that the degree of vessel density in the central part of tumor and peripheral area (invasive compartment) has a great clinical significance [15]. The degree of MVD in tumor is currently widely assessed by CD31 protein expression. This protein is a marker that can selectively detect glycoprotein of 130 kDa in vascular endothelial cells, thereby contributing to the assessment of vascularization and consequently tissue vessel density [16].

A nucleolus is an organoid in an interphase cell nucleus, the site of ribosome biogenesis. An increase in the nucleolus size and ribosome biogenesis is observed in all mammalian cells stimulated for proliferation and in tumor growth [17]. At the same time, the number of highly active types of nucleoli (nucleolonemic and compact nucleoli) is increased, and the number of low active nucleoli (ring-shaped nucleoli and fibrillar centers) is decreased. One of interesting and promising methods for determining the rate at which cells do mitosis is the assessment of the number of argyrophilic nucleolar proteins (AgNORs) regulating the activity of ribosomal genes [18, 19]. The AgNOR expression depends on the cell cycle, with minimal expression in the G0 phase and the highest expression in the S and G2 phases, and the degree of this expression depends mainly on the tumor growth rate and grade. The structure and functions of interphase AgNORs are qualitatively important parameters of cell, both in normal cells and in tumor growth, which affect the clinical course of malignant tumors. The number of AgNORs during interphase is strictly connected with rDNA transcription activity and upregulates this process during continuous cell proliferation [20, 21]. Therefore, the assessment of AgNOR activity makes it possible not only to detect the fraction of proliferating cells but also to assess the rate of proliferation [22–24]. In addition, there is evidence that the number of AgNORs may be an indicator of cell maturity [25]. This conclusion is especially important for assessing the rate of neovascularization and the degree of vessel maturity in uterine LMS and for estimating the effect of this parameter on some clinical and morphological parameters, including prognosis. However, there are no data on the number of AgNORs in leiomyosarcoma microvascular endothelial cells, although the AgNOR activity in uterine smooth muscle tumors was shown to have an exponential rise from ordinary LMS to peak values in high-grade LMS, with tumor myocytes used as an object of study [26–29].

Therefore, the objective of this study was to investigate the prognostic value of MVD in uterine LMS and PA as evaluated by CD31 protein expression and AgNOR count in endothelial cells.

## 2. Materials and Methods

### 2.1. Patients

A retrospective study was performed using data on uteri removed from patients with fast growing tumors of the uterine body from 1996 to 2009 in Altai Krai of the Russian Federation. The clinical picture included pain, bleeding, and twofold or more increase in tumor size as shown by ultrasound within a year. In LMS cases, extirpation of the uterus and its cervix and appendages was performed most often (86.4%). The study sample comprised 66 cases with uterine LMS in surgically treated patients with known prognoses. Mean age of LMS patients was 52.1 ± 1.3 years (min 23; max 77; mode 50). Almost 80% of LMS patients were pre- and postmenopausal women. The rest part of patients was reproductive-aged women, who were operated in the second half of their menstrual cycle. Single tumor nodes were observed in 89.4% of cases; multiple tumor nodes in 10.6% of cases. Tumor grade was determined using 3-grade system (FNCLCC grading system proposed by the National Federation of French Cancer Centers) correlating with survival of patients with sarcomas of different locations, which is based on 3 parameters: tumor differentiation, mitotic activity, and tumor necrosis, with summing the scores obtained for each of these 3 parameters [30]. FIGO classification was used for staging tumors.

A radical operation (extirpation of the uterus and appendages) was performed in most patients (*n* = 57; 86.4%), and the supravaginal amputation of the uterus and appendages was performed in the rest of patients, often of reproductive age (Table 1). Tumor size varied from 1 to 19 cm and was, on average, 7.3 ± 4.4 cm. Postoperative chemoradiotherapy was performed in 56% of cases. Adriablastin and cyclophosphane were most often used for chemotherapy; hormone therapy was used in two cases only. Postoperative tumor recurrence was observed in 13 (19.7%) patients. One (7.7%) patient developed LMS recurrence in the small pelvis and parietal and visceral peritoneum; 4 (30.8%) patients had metastases to the pelvic, paraaortic, and mesenteric lymph nodes; 8 (61.5%) patients had delayed metastases to the lungs. Recurrence and/or metastases occurred at a mean follow up of 3 to 4 years. To perform a comprehensive assessment of prognostic factors, all clinical characteristics included into Table 1 were further assessed using a multivariate analysis of survival criteria.

### 2.2. Tissue Specimens

Tumor specimens were taken from the central and peripheral parts of tumors. In addition, the PA (not more than 0.5 cm from a tumor) was examined in each case. Specimens were fixed in 4% neutral formalin not later than 1 hour after extirpation of the uterus. Histological techniques included hematoxylin and eosin staining and Van Giesons's method for collagen fibers.

### 2.3. Immunohistochemical Reaction for CD31

After fixation in 4% formalin buffer solution, tissue samples were embedded in paraffin, and 4 *μ*m thick sections were placed on poly-L-lysine-coated glass slides and were dried at 55°C for 35 min in a thermostat. Then the sections were cleared from paraffin with 2 changes of toluene for 5 min at room temperature and rehydrated in a graded series of ethanol (96%–70%). Further stages were performed at room temperature in a wet chamber. To unmask the antigens, we used a water bath for 35 min (BULL1000MX, Biocare, USA). The endogenous peroxidase was blocked with 3% hydrogen peroxide for 5 min. TRIS buffer was used before and after blocking procedure (4 Helendahel vials for 3 min) and then after each subsequent procedure. For immunohistochemical assay, anti-CD31 antibodies (mouse monoclonal, clone JC70A, DAKO) (not more than 1 : 10 dilution) were used. The preparations were incubated for 30 min. At the final stage, Super Sensitive Polymer-HRP Detection System/DAB (BioGenex) was used with DAB chromogen and further counterstaining of nuclei with Meyer's hematoxylin. Vascular endothelial cells that stained positively for CD31 were counted. To count MVD, a quantitative estimation was performed in absolute values at an area of 0.73 mm² in a ×200 field (not less than 3 fields were estimated, and average counts of the three fields were recorded), as proposed by Matsuyama et al. and Bosari et al. [14, 31]. For this purpose, an area with maximum concentration of vessels was identified at low magnification (×40; ×100). Then MVD was counted at a greater magnification ×200 (×20 objective lens and ×10 ocular lens; 0.73 mm² per field). Not less than three fields were examined. All vessels (both mature and immature) positive for CD31 were counted. Because CD31 can be found on macrophages, osteoclasts, platelets, and some cells engaged in immunopoiesis, to prevent diagnostic mistakes, we assessed only vascular structures with distinct (slot-like, tubular, or polymorphous) lumens and with positively stained suprabasal confluent endotheliocyte layers. Single positively stained mesenchymal smooth muscle cells were not counted. Large vessels with hyalinized walls were not counted. Skin capillary hemangioma served as a positive control. Results were assessed by two independent pathologists.

### 2.4. AgNOR Counting

AgNORs of human endotheliocytes were stained with 50% AgNO_3_ solution (Bio-Optica, Milan) according to Howell and Black, 1980 [32], as modified by Korneyev et al., 2000 [33]. The main difference of this modification is that before silver staining of preparations, the latter were treated with 2N formic acid solution for 20 min to reduce deposition of silver outside nucleoli and facilitate the count of argyrophilic granules. After silver staining, the preparations were thoroughly rinsed in distilled water, counterstained in 0.1% methyl green water solution for 20 s, and mounted in polystyrene. In each case, a mean number of nucleoli and silver grains per 1 endotheliocyte nucleus were counted in not less than 120 cells.

## 3. Statistical Analysis

The cumulative proportion of 10-year survival was estimated using the Kaplan-Meier method [34]. To assess the prognostic value of MVD, number of AgNORs of vascular endothelial cells in the PA and LMS, patient's age, and tumor size, threshold values representing mean values of indicators under study were used. This method has been proven to be effective for assessing molecular biological criteria of survival (ploidy, number of AgNORs in tumor cells, Ki-67, and p53) for some tumor locations: breast cancer, colorectal cancer, rhabdomyosarcoma, and LMS of uterus body [35–38]. Analysis of differences between survival curves was performed. For statistical hypothesis testing of mean values, the following nonparametric tests were used: Kruskal Wallis one-way test, Mann-Whitney *U* test, Kolmogorov test, and Spearman's rank correlation coefficient (*r*). Differences were significant at *P* < 0.05. Statistical processing was performed using software package STATISTICA 6.0.

## 4. Results

### 4.1. AgNOR

**Figure 1:**
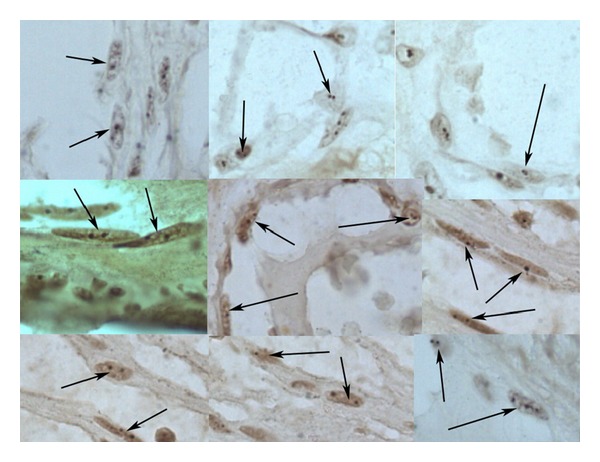
The number of AgNORs of vascular endothelial cells in native myometrium, as estimated using AgNO_3_. The number of argyrophilic granules in endotheliocyte nuclei from different areas of microvessels is not more than 2-3 granules per 2 ring-shaped nucleoli. The arrows are pointing at endothelial cells with argyrophilic granules.

Our findings showed that peritumoral tissues were characterized by modules of mature microvessels with terminally differentiated endothelial cells and a small number of AgNORs. Tumor vessels were most often immature capillary and postcapillary microvessels with immature fast-growing endothelial cells and a large number of AgNORs. The number of AgNORs of vascular endothelial cells in the PA was low (1.85 ± 0.1), with predominance of ring-shaped nucleoli (Table 1, Figures 1 and 3(a)).

**Figure 2:**
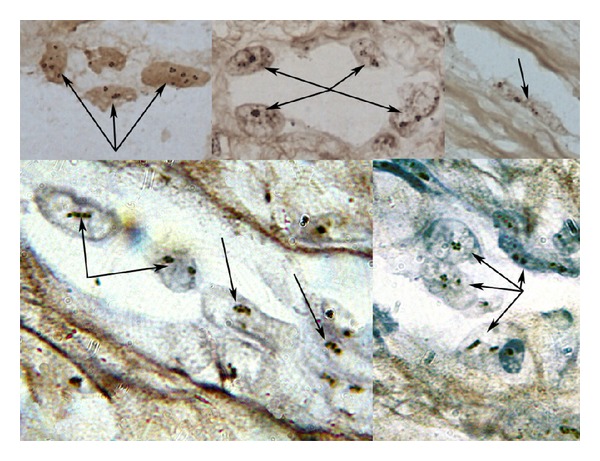
The number of AgNORs of vascular endothelial cells in sarcoma, as estimated using AgNO_3_. The number of argyrophilic granules in endotheliocyte nuclei from different areas of microvessels is more than 2-3 granules per 2 ring-shaped nucleoli. The arrows are pointing at endothelial cells with argyrophilic granules.

**Figure 3:**
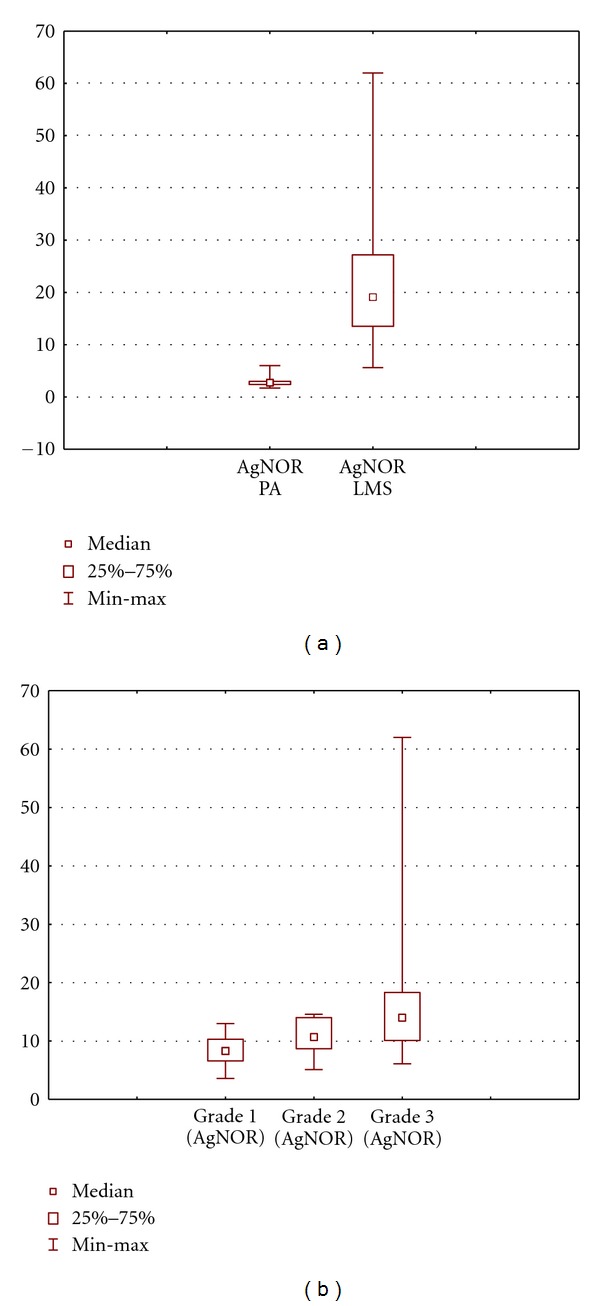
The number of AgNORs of endothelial cells in the PA and LMS: (a) total and (b) at different grades of malignancy of LMS. Data are presented as median/quartile/range.

At the same time, the number of AgNORs of vascular endothelial cells in tumor was significantly higher than that in the PA (1.85 ± 0.1 versus 11.6 ± 3.4), and more active nucleolonemic nucleoli began to dominate (Table 2, Figures 2 and 3(a)). Note that as the tumor grade increased, the number of AgNORs in endothelial cells increased from 8.3 ± 5.3 granules at grade 1 phase to 16.7 ± 4.8 granules in high-grade sarcoma (Table 2, Figures 2 and 3(b)).

There was a moderate correlation between the tumor grade and the number of AgNORs of vascular endothelial cells in tumor (*r* = 0.36; *P* = 0.03). There was no correlative relationship of the number of AgNORs of vascular endothelial cells in the PA with the tumor grade and the size of tumor node (*r* = 0.21; *P* = 0.1; *r* = 0.22). In addition, we found no dependence of the number of AgNORs of endothelial cells in the tumor and in the PA on the number of tumor nodes and the tumor stage.

### 4.2. MVD (CD31)

**Figure 4:**
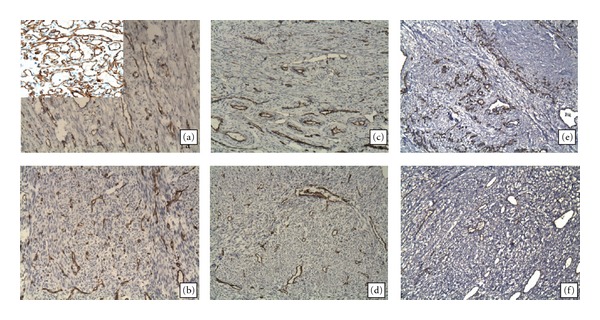
MVD (as evaluated by CD31 expression) in LMS vessels ((b), (d), (f)) and peritumoral area ((a), (c), (e)). Immunohistochemistry technique, magnification ×200. There were no significant differences in the MVD between LMS and the PA. MVD in LMS at grade 1 phase (b) was higher than at grade 2 and grade 3 phases; however, in each tumor case, indices varied in the same manner as the MVD in the PA. A positive control for CD31 (capillary hemangioma, ×400) is shown in the left upper corner.

**Figure 5:**
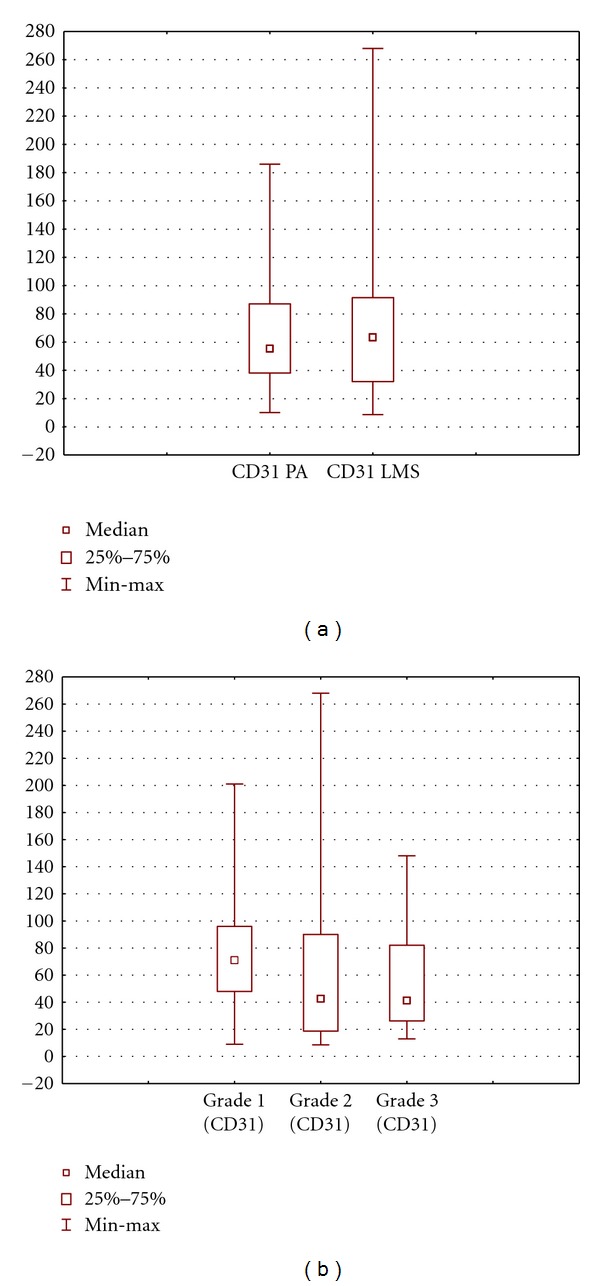
MVD in the PA and LMS: (a) total and (b) at different grades of malignancy of LMS. Data are presented as median/quartile/range.

The MVD in the PA was on average rather lower than vessel density in the tumor (64.4 ± 4.4 versus 68.8 ± 11.4), although the differences were nonsignificant and with a marked statistical excess (*P* = 0.9; excess = 2.3; Table 2, PA: Figures 4(a), 4(c), and 4(e); tumor: Figures 4(b), 4(d), 4(f), and 5(a)). We found no correlation or relationship of the MVD with the stage and grade of LMS (*r* = 0.1). For example, the MVD was 79.3 ± 8.5 in low-grade LMS and was 59.1 ± 9.1 in high-grade LMS; differences were nonsignificant (Table 2, Figures 4(b), 4(d), 4(f), and 5(b)). We also found no correlative relationship between the MVD in both tumor and peritumoral tissue and the tumor size (*r* = 0.13 and 0.21, resp.).

### 4.3. Prognosis

**Figure 6:**
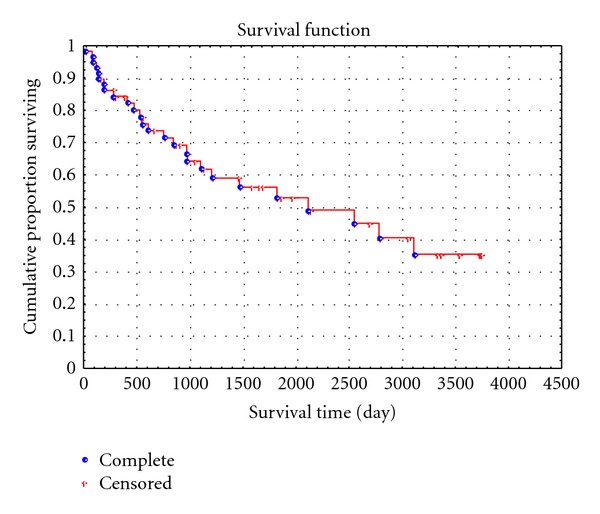
Cumulative proportion of 10-year survival of LMS patients.

Analysis of the effect of MVD in tumor and peritumoral tissue on the survival of patients with uterine LMS is of special interest. For calculations, mean (threshold) values were used for parameters under study: 1.85 (1.9) and 11.6 for AgNOR proteins of vascular endothelial cells in the tumor and the PA, respectively, and 64.4 and 71.4 for the MVD in the peritumoral tissue and tumor, respectively. The Kaplan-Meier cumulative survival analysis showed that the total 5-year and 10-year survival rates, without considering tumor grade and stage, were 0.52 ± 0.06 and 0.35 ± 0.08%; respectively, median was 5.6 years (Figure 6).

**Figure 7:**
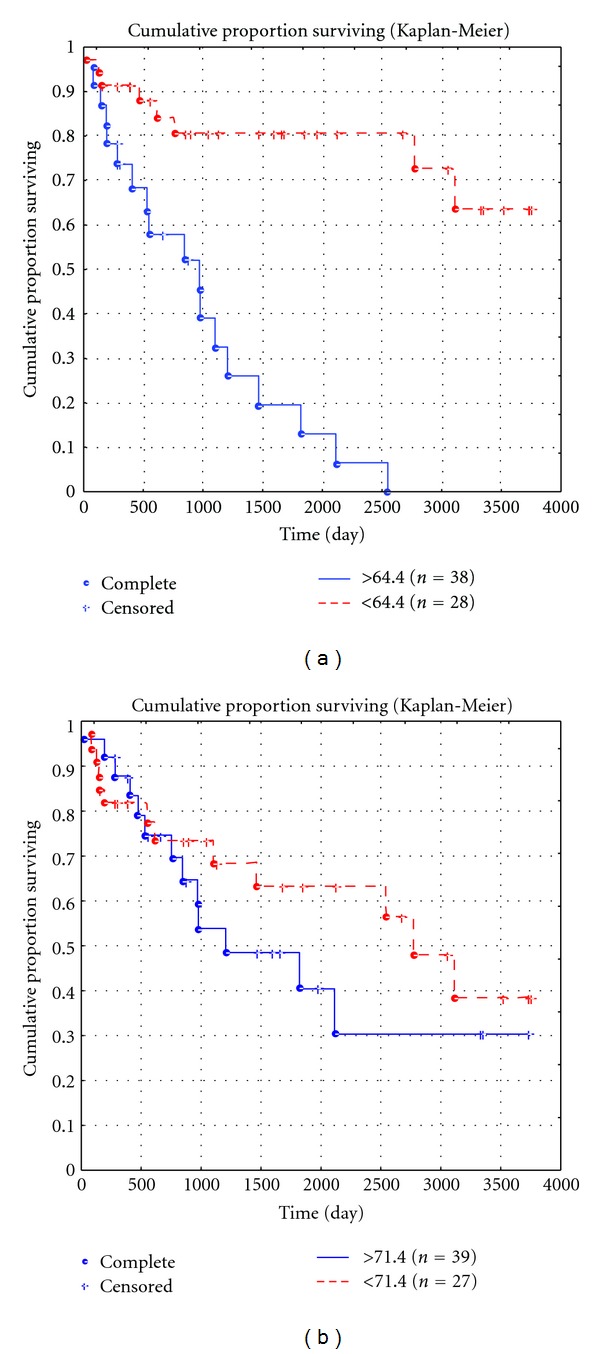
Cumulative proportion of 10-year survival of LMS patients, depending on the MVD in the PA (a) and the MVD in the tumor (b).

Our findings showed that the MVD in the PA affected the survival of LMS patients to a greater extent than the MVD in the tumor itself. For example, for the peritumoral MVD of up to threshold level of 64.4 per 0.73 mm², the 5-year survival rate was 0.79 ± 0.097%, and the 10-year survival rate was lower—0.63 ± 0.01% (Figure 7(a)).

**Figure 8:**
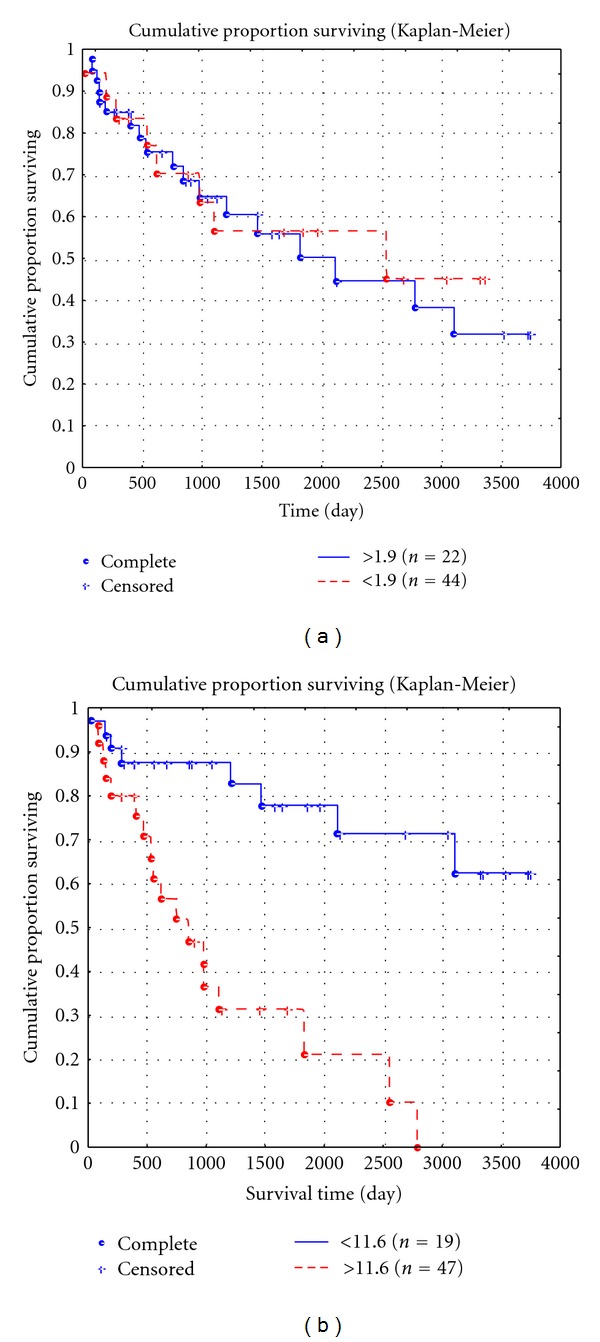
Cumulative proportion of 10-year survival of LMS patients, depending on the number of AgNOR proteins in peritumoral (a) and tumoral (b) endothelial cells.

However, when the threshold level was exceeded, the survival was significantly decreased: the 5-year survival rate was 0.20 ± 0.09%, and none of patients survived 10 years. At the same time, survival of LMS patients, depending on the MVD in the tumor itself, virtually did not differ from that both at the mean level of MVD of 71.4 per 0.73 mm² and at higher values. For example, the 5-year survival rate was 0.62 ± 0.01% at the MVD of 71.4 and was 0.39 ± 0.09% when the mean MVD level was exceeded (*P* = 0.9), and the 10-year survival rate was 0.49 ± 0.02 and 0.31 ± 0.011%, respectively (*P* = 0.9; Figure 7(b)). Analysis of the significance of the number of AgNORs for the 10-year survival showed that the 10-year survival rate was 0.44 ± 0.09% when the number of granules in vascular endothelial cells in the PA did not exceed the mean level of 1.9 and was 0.32 ± 0.073 when the mean level was exceeded; differences were nonsignificant (*P* > 0.3). At the same time, analysis of the effect of the number of AgNORs of tumoral vascular endothelial cells on patient survival showed that the 10-year survival rate was 0.62 ± 0.012% when the number of granules in tumoral vascular endothelial cells did not exceed the mean level of 11.6, and none of patients survived 10 years when the mean level was exceeded (*P* < 0.05; Figures 8(a) and 8(b)). The multivariate analysis showed that the number of AgNORs of endothelial cells in the PA and LMS had an insignificant prognostic impact, and its correlation with the 10-year survival rate was nonsignificant (*χ*_2_ values 0.01 and 1.9, resp.).

Our findings on the prognostic value of the number of AgNORs of vascular endothelial cells in the tumor and the MVD in the PA are rather interesting and controversial. A comparison of survival curves depending on these indices showed rather similar results. In fact, for the peritumoral MVD of up to threshold level, the 5-year survival rate was 0.79 ± 0.097, and the 10-year survival rate was 0.63 ± 0.01, and when the threshold level was exceeded, the 5-year survival rate was significantly lower (0.20 ± 0.09), and none of the patients survived for 10 years (Figure 7(a)). This was consistent with the number of AgNORs of endothelial cells in the tumor: for up to threshold level, the 5-year survival rate was 0.69 ± 0.077 and the 10-year survival rate was lower (0.62 ± 0.012%), and when the threshold level was exceeded, the 5-year survival rate was 0.31 ± 0.08, and none of the patients survived for 10 years (Figure 8(b)). Thus, we found rather different survival indicators with comparable tendencies. However, a further multivariate analysis did not show high *χ*_2_  values for the number of AgNORs of vascular endothelial cells in the tumor, which might be explained by a close correlation of this indicator with the proliferative activity of tumor cells (at *r* = 0.58), whereas in the univariate analysis of the prognostic value of indicators, this indicator had rather high values (*Z* = 1.5), although lower than those for the MVD in the PA (*Z* = 3.3), but these tendencies need further investigation.

In a Cox regression multivariate analysis of microvasculature development, the MVD in the peritumoral area was the only independent prognostic indicator among the factors that were assessed to determine an independent factor of 10-year survival (Table 3).

**Figure 9:**
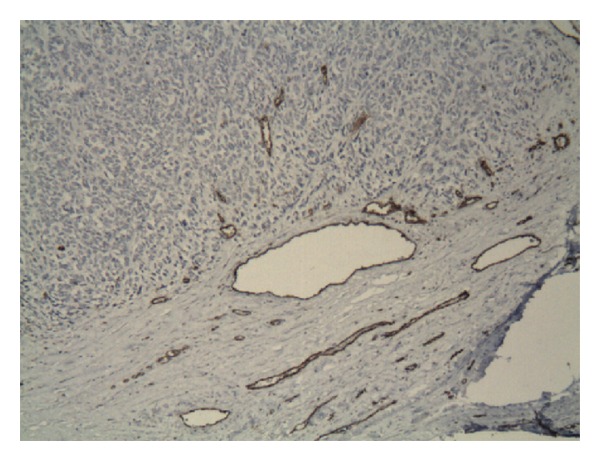
Patient N. aged 57 years, low MVD in the tumor and high MVD in the PA; died within 2 years after surgical operation.

This pattern was expressed as follows: even with a low MVD in the tumor but a high MVD in the PA, tumor prognosis was significantly more unfavorable than that in the reverse situation (Figure 9).

## 5. Discussion

Today there are many studies that focus on neoangiogenesis and MVD in tumors of different locations. Tumor angiogenesis and MVD were shown to result in the rapid tumor progression and increased invasivity and metastatic activity. The relationship to metastasis was found in non-small-cell lung cancer [14] and breast carcinoma [39, 40]. According to data reported by some investigators [41, 42], angiogenesis is an independent prognostic factor in breast carcinoma and prostate cancer. However, despite certain progress in this area, there are many unsolved questions regarding not only the role of vessels in tumor progression but also the significance of peritumoral MVD and the effect of this factor on patient survival in some malignant neoplasias [12–14].

Several changes have recently been detected in morphologically intact tissues, tumor field area, or PA [43, 44]; however, data on angiogenesis in this area are few and contradictory.

Our findings showed that MVD did not depend on the tumor stage, size, and grade in uterine LMS. Moreover, we found no significant differences or correlation in the MVD between the tumor and the PA, and statistical dispersion was significant in both the tumor and the PA. In some cases, there were tumors with a marked MVD in the tumor and low MVD in the PA and with a great number of vessels in the PA and a small number of vessels in the tumor. The number of AgNORs of endothelial cells in the tumor and the PA was also not associated with the tumor size and stage, although there was a clear relationship with the tumor grade (*r* = 0.36).

Correlation between the degree of angiogenesis and MVD and proliferative activity of endothelial cells remains incompletely understood [40], and correlation with vessel growth rate has been poorly studied. We accept the opinion that the number of AgNORs is an indicator of cell division rate and consequently cell growth rate [45–48] and consider it possible to conclude that the number of AgNOR granules in endothelial cells reflects the endothelial proliferation rate. Our findings are in line with data reported in [49], which showed that endothelial cells of terminally differentiated microvessels usually had 1-2 ring-shaped nucleoli with single large fibrillar centers, and immature proliferating endotheliocytes contained large nucleolonemic nucleoli with multiple fibrillar centers. Our findings indicate the relationship of the MVD and proliferation rate in LMS with some clinical morphological parameters, including prognosis. The mean number of AgNORs in endothelial cells was reflected during analysis of survival of patients with uterine LMS, when the increased vessel growth rate (even without a marked density of tumor vessels) was associated with a decreased 10-year survival. According to the data in the literature, the number of AgNORs has been proven to be a prognostic factor for malignant neoplasms of different locations: in Cox multivariate analysis, the number of nucleolar organizer regions and the histological type were the main prognostic factors predicting the outcome in laryngeal cancer, and their prognostic value was higher than that of Ki-67 expression [50]. Interestingly, on the one hand, the authors showed that the number of AgNORs was an independent prognostic factor in malignant tumors of different locations: oropharyngeal cancer (variation 7.13–16.1; mean 11.1), multiple myeloma (variation 2.2–9.9; mean 4.4.), malignant thymoma (variation 3.2–12.2; mean 5.6), breast cancer (variation 4.5–13.5; mean 7.4), prostate carcinoma (variation 4.1–13.4; mean 7.8), and colorectal cancer, and on the other hand, the variation in the number of AgNORs was very high for tumors of different locations [37, 51]. In our previous papers, the multivariate analysis of survival criteria showed a high prognostic value of the number of AgNORs in uterine LMS cells (*χ*² = 13.4); however, we did not assess the number of AgNORs in vascular endothelial cells [52].

In addition, we find that the number of AgNORs of vascular endothelial cells in the tumor itself is higher than that in the peritumoral area, which may be indirectly explained by the MVD. However, both the MVD and the number of AgNORs are qualitatively different indicators that do not always show concordant increases or decreases. A large number of AgNORs of vascular endothelial cells in the tumor to some extent reflect the rapid vascular growth, and the MVD is the final stage of angiogenesis. Therefore, we looked for higher MVD values in the tumor, as compared to the PA, but with a substantial spread of mean values. In some cases, with the large number of AgNORs of vascular endothelial cells in LMS, the MVD in the PA was higher than that in the tumor itself. This could be explained by the fact that, in certain cases, a high MVD in the PA is constitutional (physiological) rather than a result of angiogenesis. In these cases, the number of AgNORs of vascular endothelial cells in the PA was not large. Because a large number of AgNORs and consequently a high activity of AgNORs indicate the immaturity of both tumor and normal cells (e.g., differentiated gastric epithelial cells in the regenerative mucosa), we suggest that the high activity of proteins in endothelial cells in LMS may indicate their immaturity. A low activity of proteins in vascular endothelial cells in the PA may indicate their maturity. Therefore, we may suggest that, in some cases, the high MVD in the PA indicates a physiological condition characteristic of a certain individual rather than a marked angiogenesis. Considering that peritumoral vessels are module-type vessels, with a low activity of AgNORs in mature endothelial cells, we may suggest that peritumoral angiogenesis is rare or mild, and the MVD is a constitutional feature that is virtually unchangeable. Our findings are consistent with those of Hollemann et al., 2012, as to the fact that high vessel density, cell immaturity, and structurally abnormal vessels with barely any activity were found in tumor tissue, whereas the peritumoral tissue vessels showed a mature architecture with tight endothelial cell-pericyte interaction; however, our findings are not consistent with the fact that peritumoral tissue vessels showed a high activity of angiogenesis [53]. Our argument is that, with high angiogenesis, the normal module-type architecture will have no time to form, and high expression of CD133/VEGFR2 may simply reflect the cell receptor status. Therefore, instead of high angiogenesis in the PA, we speak about the variation in the MVD in this area, with the MVD higher in the PA as compared to the tumor itself in some cases, which led to an unfavorable outcome. Our findings are consistent to some extent with those of Al-Najar et al., 2012, who showed that survival of 61 patients with squamous cell carcinoma of the penis directly depended on the peritumoral MVD and did not depend on the tumor MVD [54]. According to their findings, a high peritumoral MVD, compared to low peritumoral MVD, was associated with a better 5-year overall survival (75% and 30%, resp.). Our findings were quite the opposite: a high peritumoral MVD, compared to low peritumoral MVD, was associated with a significantly poorer prognosis. At the same time, we agree with the opinion on a statistical excess of MVD in the PA and the tumor itself, which varies individually and separately in each case. In addition, our findings are comparable with those of Poncelet et al., 2004, who detected a relatively larger MVD in normal myometrium, as compared to the MVD in LM and LMS, although with significant variation of values (107.0 ± 53.6, 66.2 ± 55.4, and 64.4 ± 44.2, resp.) by the level of CD34, CD31, and VEGF expression [55]. At the same time, the authors believe that increased angiogenesis in LMS rather than the PA correlated with the tumor recurrence and prognosis. This is rather inconsistent with our opinion.

The number of AgNORs in the nucleus reflects not only the rate at which cells do mitosis but to a greater extent the cell proliferation rate. Correlation between Ki-67 and AgNOR expression was shown in several papers. For example, in breast cancer samples double-stained for Ki-67 and AgNOR, the number of argyrophilic granules in nuclei was higher in cells with Ki-67 expression, compared to cells without Ki-67 expression. A high correlation with the number of AgNORs and the granule area was observed (*r* = 0.53, *P* ≤ 0.01) [56]. For cell cultures with a high correlation between MIB-1 and AgNOR expression, a conclusion was made that a combination of these two markers, with the calculation of the proliferating cell pool, will be useful for measuring the cell proliferation rate in normal and malignant tissues [24]. The same conclusion on the correlation between AgNOR and other markers of cellular proliferation (MIB-1, PCNA, BrdU-labeling, and rRNA transcription activity) was made by another group of researchers [57]. When analyzing the prognostic value of Ki-67 in LMS, the authors concluded that this marker alone could be used as a survival indicator in LMS patients, although combination of tumor size, mitotic index, and Bcl-2 and CD163 expression worked even better [58]. In our work, we show the necessity to search for combined survival models using several markers to give an individual prognosis for LMS.

Notably, the peritumoral MVD affected the survival of LMS patients to a greater extent than the MVD in the tumor itself. At first sight, these findings may seem paradoxical; however, in our opinion, they indicate the significance of angiogenesis in the PA. In our opinion, peritumoral vascular modules have a more functional structure and are not excluded from circulation in the organ (in our situation, the uterus) and the whole body, while tumor vessels are less suitable for supplying the tumor and metastatic spread of tumor. It is therefore likely that tumor cells stimulate angiogenesis in the PA, and invasion of vessels, with usually subsequent metastatic spread, more often occurs at the tumor periphery.

Thus, analysis of prognostic value of MVD in uterine LMS and PA showed that the tumor vessel density was not an independent criterion, while peritumoral MVD affected 10-year survival to a significantly greater extent. These data may have not only theoretical but also practical value. Today clinical repertoire includes antiangiogenic therapy (including targeted therapy, e.g., Avastin) to inhibit tumor neoangiogenesis and probably peritumoral vessels in LMS, as compared to vessels of the tumor itself, must be exposed to antiangiogenic therapy to a greater extent. However, this idea needs further thorough investigation. 

## Figures and Tables

**Table 1:** Clinical characteristics of patients under study.

Clinical characteristic	Index
Stage according to FIGO classification	
I	*n* = 43 (65.2%)
II	*n* = 23 (34.8%)
Age	
<50 years	*n* = 26 (39.4%)
50 years	*n* = 40 (60.6%)
Type of surgery	
Supravaginal amputation of the uterus and appendages	*n* = 9 (13.6%)
Extirpation of the uterus and appendages	*n* = 57 (86.4%)
Tumor size	7.3 ± 4.4 (min: 1, max: 19, moda: 3)
Postoperative chemoradiotherapy	*n* = 37 (56.1%)
Recurrence and/or metastases	*n* = 13 (19.7%)

**Table 2:** The number of AgNORs of endothelial cells and the MVD in the PA and LMS of different stages and grades of malignancy.

Object of study		CD31	AgNORs of endothelial cells
PA (*n* = 66)		64.4 ± 4.4	1.85 ± 0.1^∗^
		(min—10; max—104)	(min—1; max—3)

Sarcoma of different grades of malignancy	G1	79.3 ± 8.5	8.3 ± 5.3^#^
	(*n* = 30)	(min—9.0; max—201)	(min—3; max—13)
	G2	68.2 ± 16.6	9.8 ± 1.5
	(*n* = 17)	(min—8.6; max—268)	(min—2; max—14)
	G3	59.1 ± 9.1	16.7 ± 4.8^#^
	(*n* = 19)	(min—13; max—148)	(min—5; max—62)

Sarcoma of different stages	I	80.7 ± 8.3	10.3 ± 1.5
	(*n* = 43)	(min—9; max—268)	(min—3; max—32)
	II	52.2 ± 8.7	13.5 ± 4.3
	(*n* = 23)	(min—8.6; max—148)	(min—5; max—62)

*Differences in the number of AgNORs of endothelial cells in the PA and LMS at *P* ≤ 0.05.

^#^Differences between groups of different grades of malignancy at *P* ≤ 0.05.

**Table 3:** Significance of prognostic criteria as evaluated by Cox regression multivariate analysis.

Clinical and pathological feature	*χ* ^2^	*P*
Stage (according to FIGO)	9	0.002
pTNM category	10.9	0.0009
Age	6.5	0.01
Type of surgery	0.2	0.8
Tumor size	0.17	0.7
Chemoradiotherapy	2.3	0.1
Recurrence/metastases	9.6	0.02
Grade of malignancy	14.2	0.0002
AgNOR in peritumoral area	0.01	0.9
AgNOR in tumor	1.9	0.1
MVD (CD31) in peritumoral area	27.5	0.0003
MVD (CD31) in tumor	0.1	0.8
